# Mechanical gating of a mechanochemical reaction cascade

**DOI:** 10.1038/ncomms13433

**Published:** 2016-11-16

**Authors:** Junpeng Wang, Tatiana B. Kouznetsova, Roman Boulatov, Stephen L. Craig

**Affiliations:** 1 Department of Chemistry, Duke University, Durham, North Carolina 27708, USA; 2 Department of Chemistry, University of Liverpool, Crown Street, Liverpool L69 7ZD, UK

## Abstract

Covalent polymer mechanochemistry offers promising opportunities for the control and engineering of reactivity. To date, covalent mechanochemistry has largely been limited to individual reactions, but it also presents potential for intricate reaction systems and feedback loops. Here we report a molecular architecture, in which a cyclobutane mechanophore functions as a gate to regulate the activation of a second mechanophore, dichlorocyclopropane, resulting in a mechanochemical cascade reaction. Single-molecule force spectroscopy, pulsed ultrasonication experiments and DFT-level calculations support gating and indicate that extra force of >0.5 nN needs to be applied to a polymer of gated *g*DCC than of free *g*DCC for the mechanochemical isomerization *g*DCC to proceed at equal rate. The gating concept provides a mechanism by which to regulate stress-responsive behaviours, such as load-strengthening and mechanochromism, in future materials designs.

## Abstract (web summary)

Polymer mechanochemistry offers opportunities to control and engineer desired chemical transformations. Here, Craig and co-workers present a mechanical gating system whereby one mechanophore modulates the reactivity of another, resulting in a mechanochemical cascade reaction.

## Graphical abstract

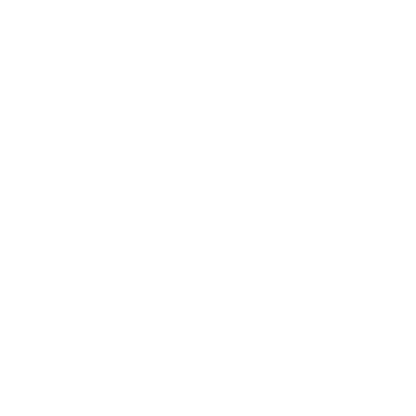

Covalent polymer mechanochemistry^1^,^2^,^3^,^4^,^5^ has been extensively explored in recent years for a variety of purposes, including biasing reaction pathways^6^,^7^,^8^,^9^, trapping transition states and intermediates^10^,^11^, catalysis^12^,^13^,^14^,^15^, release of small molecules and protons^16^,^17^,^18^, stress reporting^19^,^20^,^21^,^22^, stress strengthening^22^,^23^,^24^ and soft devices^25^,^26^. These efforts are largely facilitated by the fact that, unlike other energy sources, mechanical force is directional and regulated by local molecular structure. Even reactions with very similar intrinsic activation barriers can have very different force-coupled reactivities as a result of the structure of the handle through which force is delivered^3^,^8^,^27^,^28^,^29^.

Both experimental^6^,^7^,^24^,^27^,^30^,^31^,^32^,^33^,^34^,^35^,^36^,^37^,^38^ and theoretical^29^,^39^,^40^,^41^,^42^,^43^,^44^,^45^,^46^,^47^,^48^ studies have been conducted to understand force-coupled reactivity and mechanisms, and these investigations provide valuable insights into the design of mechanophore response. One concept for a useful reactivity response is mechanochemical gating, in which one mechanophore (a molecular gate) initially prevents another mechanophore (substrate) from experiencing force delivered along a polymer backbone. When the gate is unlocked mechanically, the substrate feels force and reacts at once in a new type of mechanochemical cascade reaction, reminiscent of the allosteric gating in biological ion transport and signal transmission^49^,^50^,^51^, but driven mechanically rather than by voltage or ligand coupling^49^,^50^,^51^.

Here, we illustrate the gating concept by demonstrating force-gated isomerization of *cis*-*gem*-dichlorocyclopropane (*cis*-*g*DCC) by cyclobutane in a dual mechanophore, 5,5-dichlorotricyclo(7.2.0.0)undecane (DCTCU). Single-molecule force spectroscopy and ultrasonication experiments reveal that a larger force needs to be applied to a polymer of DCTCU than the polymer of *cis*-*g*DCC to isomerize *cis*-*g*DCC to dichloroalkene at the same rate. Quantum-chemical calculations confirm that the difference reflects the additional force needed to open the cyclobutane core, that is, that the mechanochemical kinetics of *cis*-*g*DCC isomerization is controlled by that of cyclobutane dissociation rather than the intrinsic mechanochemistry of *cis-g*DCC.

## Results

### Computational predictions

**Figure 1:**
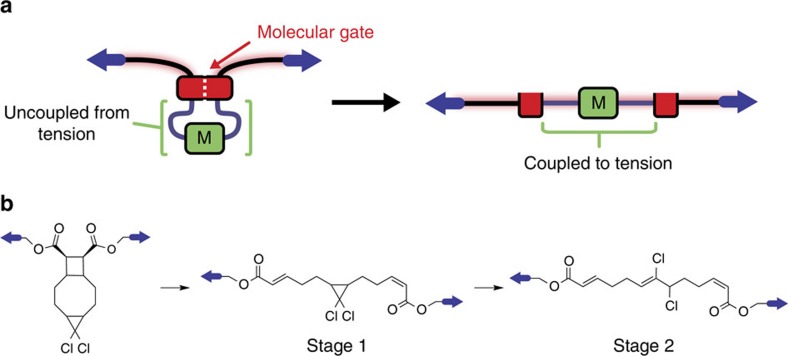
Schematic representation and molecular design of mechanical reaction gating. (**a**) A molecular gate (red block) prevents a protected mechanophore substrate (green) from feeling force. Applying sufficiently high force to the gate unlocks it mechanically, allowing the force to be transmitted to the substrate. (**b**) A cyclobutane mechanophore functions as the gate, and its mechanical cycloreversion unlocks the system so that initially protected *cis*-*gem*-dichlorocyclopropane (*cis-g*DCC) is activated.

**Figure 2:**
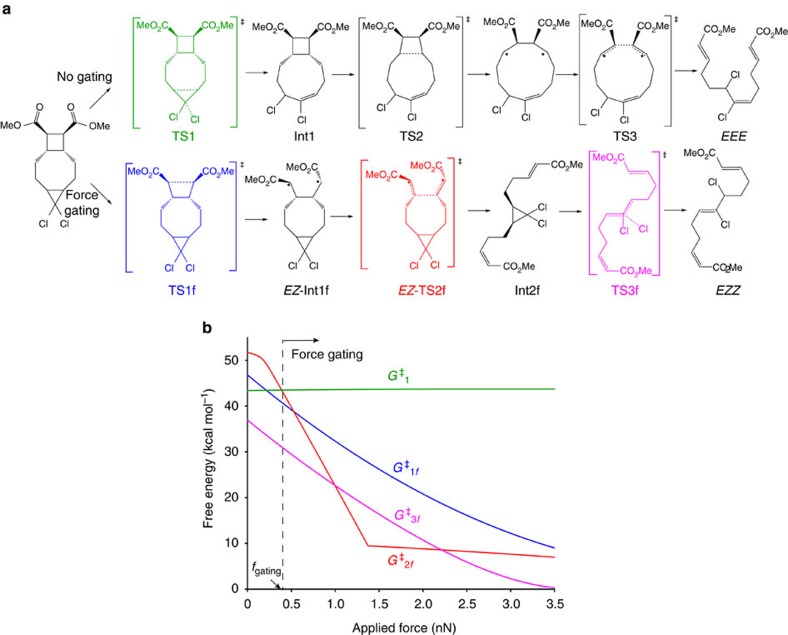
The computed effects of force on the reaction mechanism and activation barriers. (**a**) The minimum-energy reaction mechanisms without and with gating are different as are the final products. The relative contributions of the *EE* and *EZ* conformers of Int1f and TS2f vary with force (Supplementary Fig. 2): for clarity only one set of isomers is shown. (**b**) Calculated heights of the free energy barriers (see text for assumptions and details of theoretical methods) in methylbenzoate as a function of the applied force calculated as the differences of the free energies of (i) TS1 and TS1f relative to 5,5-dichlorotricyclo(7.2.0.0)undecane (DCTCU) (*G*_1_ ^‡^ and *G*_1*f*_ ^‡^, respectively), and (ii) TS3f relative to Int2f (*G*_3*f*_ ^‡^); *G*_2*f*_ ^‡^ is the free energy of TS2f relative to (i) DCTCU up to 1.4 nN and (ii) Int1f at >1.4 nN, when Int1f becomes lower in energy than DCTCU. The calculated cross-over between the non-gated and gated reactivity, *f*_gating_, occurs at ∼0.45 nN as indicated.

The gating concept and our initial system are shown in Fig. 1. Calculations at the uMPW1K/6-311+G(d) level of DFT with a polarized-continuum model of reaction solvent called CPCM (ref. 52) (used to account for observed solvent effects, Supplementary Table 1) reveal several mechanisms for dissociation of the three scissile bonds of DCTCU (Supplementary Figs 1 and 2) whose relative importance changes with applied force. In the absence of force, the most reactive site of DCTCU is *cis*-*g*DCC, whose opening results in a bicyclic intermediate Int1 (Fig. 2a), followed by sequential homolysis of the inner and then of the outer C–C bonds of cyclobutane to yield *EEE*-triene.

Stretching force acting on the C atoms of the CH_3_ groups of DCTCU does not affect the apparent activation energy of this path (green line, Fig. 2b), but lowers all barriers of an alternative isomerization mechanism in which the order of dissociation of the 3 scissile bonds is inverted (Fig. 2a). This inversion is a result of the sequential loading of the scissile bonds enforced by the fused-ring architecture of DCTCU (and its homologues): the applied force is coupled to only one scissile bond at a time, which must dissociate before another scissile bond is loaded. Because force strongly accelerates bond dissociation, this sequential loading ensures that bonds break in the same order that they are loaded. For example, when stretching force of 1 nN is applied to DCTCU, the calculated activation free energy for dissociation of the outer C–C bond of the cyclobutane moiety (TS1f, blue) is reduced to 32.5 kcal mol^−1^ compared with 51.7 kcal mol^−1^ and 41.7 kcal mol^−1^ for dissociation of the bridgehead bonds of the cyclobutane and *cis*-*g*DCC moieties, respectively, which are uncoupled from the applied force (all numbers are for reaction in methylbenzoate solvent). Consequently, the reaction proceeds through TS1f, yielding intermediate Int1f, in which the applied force acting on the second scissile bond of cyclobutane reduces the free-energy barrier of its dissociation to 22.5 kcal mol^−1^ (TS2f, red). The *cis*-*g*DCC moiety in Int1f remains shielded from the force and hence its barrier unchanged at 41.7 kcal mol^−1^. Only once TS2f is traversed is the applied force transmitted to the *cis*-*g*DCC moiety, lowering its reaction barrier to 22.6 kcal mol^−1^ (TS3f, magenta).

In other words, at applied forces where the gating mechanism of Fig. 2a dominates, the rate of *cis*-*g*DCC isomerization is governed by the largest of the activation barrier for dissociation of either the outer (TS1f) or the inner (TS2f) C–C bonds of cyclobutane, rather than by the energy of TS3f. For example at 1 nN in toluene, the rate-determining kinetic barrier for gate dissociation (TS1f) exceeds that of *cis*-*g*DCC isomerization (TS3f) by 9.2 kcal mol^−1^, corresponding to a >10^5^-fold increase in the *cis*-*g*DCC half-life at room temperature. Equivalently, a polymer of DCTCU would have to be stretched to a force of 1.8 nN to generate the dichloropentene moiety at the same rate as would poly(*cis-g*DCC) stretched to 1 nN.

### Single-molecule force spectroscopy

**Figure 3:**
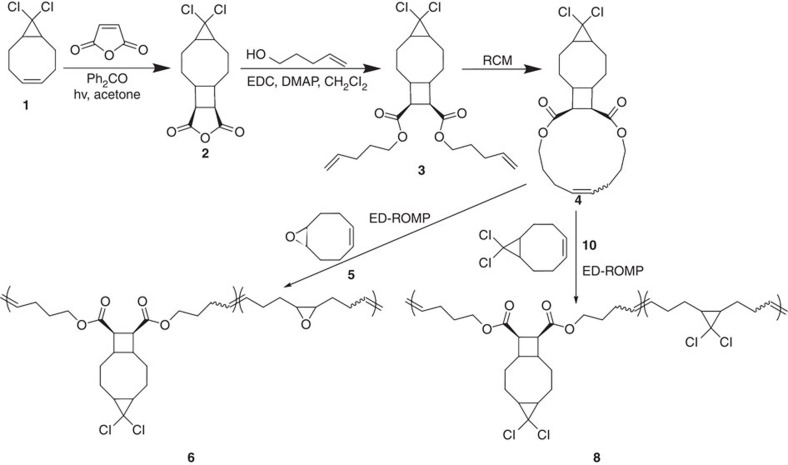
Chemical structures and synthesis of polymers 6 and 8. Experimental details are provided in the Supplementary Methods; EDC and DMAP are N-(3-dimethylaminopropyl)-N′-ethylcarbodiimide hydrochloride and 4-dimethylaminopyridine, respectively. The ring closing metathesis and entropically-driven ring opening metathesis co-polymerization steps used Grubbs second generation catalysts.

Results of both single-molecule force spectroscopy (SMFS) and sonication experiments are consistent with gated mechanochemistry of *cis-g*DCC in DCTCU. The experiments were conducted on polymers containing multiple DCTCUs in the backbone, a strategy that we used in the past to quantify mechanochemistry of *gem*-dihalocylopropanes^7^,^27^,^33^, benzocyclobutenes^7^ and spiropyran^53^. The synthesis (Fig. 3) was similar to that of previously reported bicyclo(4.2.0)octane derivatives^34^. Photochemical (2+2) cycloaddition of maleic anhydride and *Z*-9,9-dichlorobicyclo(6.1.0)non-4-ene (**1**) in the presence of benzophenone yielded DCTCU derivative (**2**), which was esterified with 4-pentenol to diene **3** followed by ring closing metathesis^54^ to form macrocycle **4**. Entropically-driven ring opening metathesis co-polymerization^55^,^56^,^57^ of **4** with either unsaturated epoxide **5** or *cis*-*g*DCC derivative **10** yielded polymer **6** and **8** for SMFS and sonication experiments, respectively. We incorporated epoxides in polymer **6** to increase its adhesion to the tip of atomic force microscope^27^. The identity of all synthetic intermediates and final products were confirmed by ^1^H and ^13^C NMR spectroscopy (Supplementary Fig. 3).

**Figure 4:**
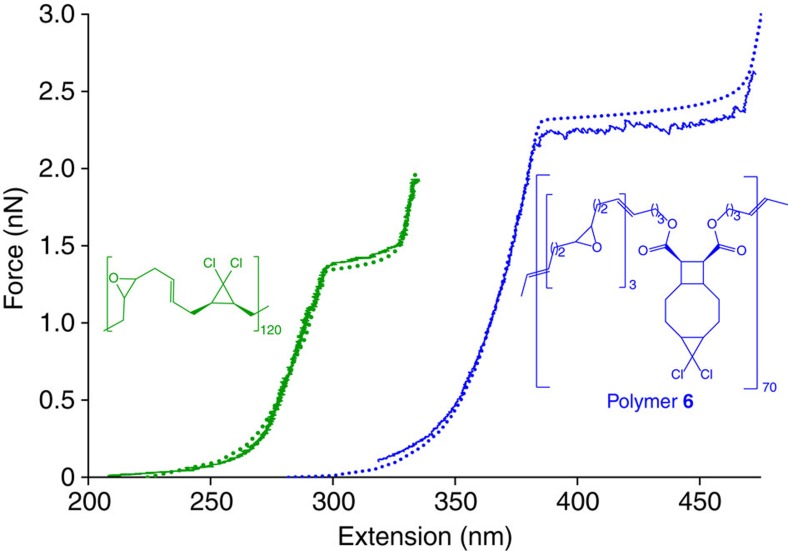
Measured and computed force–extension curves. The experimental (solid lines) and computational (dotted lines) results of a single macromolecule of polymer **6** and of a polymer of *cis*-*g*DCC are shown in blue and green, respectively. The molar ratios of epoxide/DCTCU or epoxide/*cis*-*g*DCC moieties used to calculate the force–extension curves (red and green structures) were chosen to approximate the ratios measured in bulk polymer samples by ^1^H NMR. Additional measured force–extension curves are shown in Supplementary Figs 4 and 5 and the computed curves are tabulated in Supplementary Table 3.

The SMFS measurements were conducted at a tip velocity of 300 nm s^−1^ using the previously described procedure^7^. Figure 4 shows a representative measured force–extension curve for polymer **6** and for comparison a previously reported^27^ force–extension curve of poly(*cis*-*g*DCC) along with the curves extrapolated from the force/activation energy correlations described in Fig. 2b. The average force at the middle of the plateau of the 10 force–extension curves measured was 2.2±0.15 nN, in good agreement with the predicted force of 2.3 nN at the equivalent loading rate. We observed no statistically significant dependence of the measured plateau force on the polymer length, the length of the plateau or the molar fraction of the DCTCU moieties measured on bulk samples of polymer **6** by ^1^H NMR spectroscopy (Supplementary Table 2).

The plateau regions of the force–extension curves for **6** show greater saw-tooth texture than previously reported curves of other multi-mechanophore polymers^7^,^27^,^33^,^53^, a feature that we attribute to the considerable hidden length (that is, extension generated from ring-opening) of DCTCU. We cannot resolve individual ring-opening events in the force–extension curves, but comparing the length of the plateau with the calculated hidden length of a single DCTCU moiety (1.2 nm at 2 nN) indicates that in the 10 measured force/extension curves the number of DCTCU units responsible for the observed plateau varied from 11 to 160, with the median of 46. The force over which these molecules isomerized varied from ∼70 to ∼200 pN but is uncorrelated with the number of reacted molecules, in line with the range reported for other multi-mechanophore polymers, suggesting that even as few as 10 DCTCU molecules is sufficient to approximate the behaviour of an ensemble. The ∼575 isomerization events measured in our SMF experiments all occurred between 2 and 2.4 nN, which is consistent with the calculated change in the survival probability of DCTCU under the relevant conditions decreasing from ∼0.999 to ∼0.02 over this range (the values depend slightly on the length of the polymer in each experiment). This contrasts with a recent report^58^ where a total of nine force-accelerated events, one per force–extension curve, attributed to the ring opening of a related cyclobutane derivative occurred at applied forces that were distributed almost uniformly over the 1.7–3.9 nN range. The difference cannot be ascribed to the 15-fold faster retraction rate and shorter polymers used in ref. 58, but may indicate that the rate-determining barrier of the process studied in ref. 58 is largely independent of the applied force between 1.7 and 3.9 nN, similarly to the second barrier of DCTCU isomerization (red curve, Fig. 2b). This speculation, however, remains to be tested computationally and experimentally.

The SMFS behaviour of polymer **6** is consistent with the expected gating dynamics. Under comparable conditions the mechanochemical ring-opening in non-gated poly(*cis*-*g*DCC) occurs at 1.3 nN, or at >0.8 nN lower force than the plateau of polymer **6**. This qualitative observation is further reinforced by the agreement between the measured and calculated force–extension curves for poly(*cis*-*g*DCC) and polymer **6** (Fig. 4). Both theory and experiment, therefore, support cyclobutane-gating of mechanochemistry of *cis*-*g*DCC in polymer **6.**

### Sonication experiments

**Figure 5:**
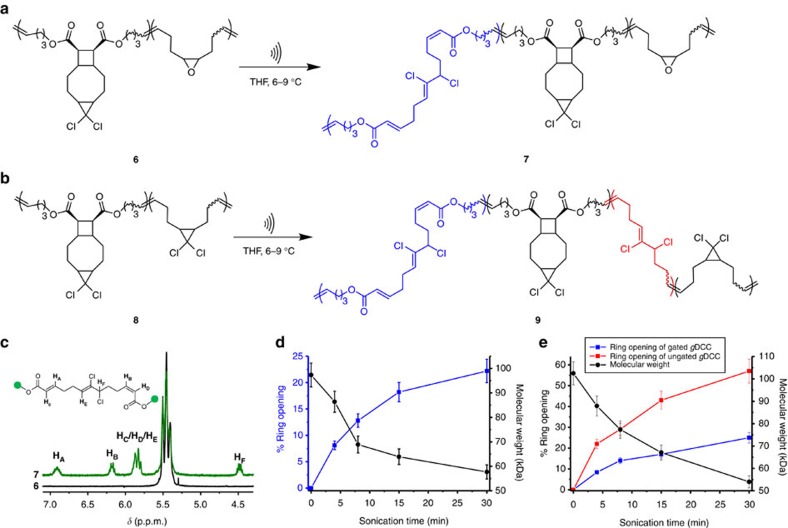
Sonication of copolymers of DCTCU with epoxide (6) and *cis*-*g*DCC (8). (**a**,**b**) Primary chemical reactions resulting from sonication of **6** (**a**) and **8** (**b**). Sonication of **8** leads to ring opening of *g*DCC gated within the initial DCTCU (blue fragment in 9) and ungated along the polymer backbone (red fragment). (**c**) Partial ^1^H NMR spectrum of polymer **6** before (black) and after (green) sonication. (**d**,**e**) Molar fractions of isomerized DCTCU moieties (blue, polymers **6** and **8**) and backbone *g*DCC moieties (red, polymer **8** only) as a function of sonication time of polymer **6** (**d**) and **8** (**e**). As is typical for every other polymer reported, non-selective backbone fragmentation competes with mechanophore-centered chemistry during sonication of polymers **6** and **8**, decreasing their average molecular weights (black axes and Supplementary Figs 8 and 9). Error bars are standard deviations.

Our QM calculations suggest that mechanical gating should be retained even at forces in the >3 nN range characteristic of polymer mechanochemistry during pulsed ultrasonication, which was indeed confirmed with the products characterized by ^1^H-NMR spectroscopy. As shown in Fig. 2b, in DCTCU, mechanochemical isomerization of *cis*-*g*DCC can only occur after complete dissociation of the cyclobutane, and the activation energy for *cis*-*g*DCC isomerization is much lower than that for the cyclobutane gating reaction at the same applied force. This means that the molar fractions of *cis*-*g*DCC and cyclobutane moieties that ring open during sonication should be identical. ^1^H NMR spectra of THF solutions of polymer **6** subjected to pulsed ultrasonication (at power density of 11.9 W cm^−2^) showed the signals of *E* and *Z* isomers of *α*,*β*-unsaturated ester (δ∼5.8, 6.2 and 6.9 p.p.m.) and of the *Z*-2,3-dichloroalkene (δ∼4.5 and 5.8 p.p.m.), expected from ring opening of cyclobutane^34^ and *cis-g*DCC (ref. 59), respectively (Fig. 5a,c; DCTCU has no peaks at these chemical shifts). Integration of the peaks (Fig. 5d, Supplementary Fig. 6) shows that the fraction of ring opening products for cyclobutane and *cis-g*DCC was identical, consistent with the expected sequential activation.

To further validate the mechanical gating mechanism, random copolymers of DCTCU with *cis*-*g*DCC (polymer **8**, Fig. 5b) were synthesized and sonicated (Supplementary Fig. 7). As shown in Fig. 5e, in contrast to the pure DCTCU polymer **6**, sonication of **8** produced ∼3.5 *cis-g*DCC ring opening events per cyclobutane scission. Since as established above this ratio is 1:1 for mechanochemical isomerization of DCTCU, the 2.5-fold excess of *cis-g*DCC ring opening reflects the higher reactivity of ungated *cis-g*DCC than the gated one, because the force-coupled reactivity of *cis*-*g*DCC during sonication is greater than cyclobutane, as predicted by our computations. The cyclobutane gate therefore determines the mechanochemical reactivity of *cis*-*g*DCC across the force range accessed in both SMFS and sonication, in both situations increasing the applied force needed to lower the half-life of *cis*-*g*DCC to the relevant timescale of each experiment.

## Discussion

The gating concept applied here is deceptive in its apparent simplicity, and we illustrate some important subtleties on the example of DCTCU. First, gating requires that dissociation of the gate traverse a higher barrier than that of the substrate (*g*DCC in our case) at the same force. Otherwise, the rate at which the substrate reacts in the loaded mechanophore will be controlled by the intrinsic mechanochemical kinetics of the substrate and will be identical to that of non-gated analogue. DCTCU meets this condition over all forces, as evidenced by the transition state of *g*DCC isomerization (magenta curve in Fig. 2b) being more stable than at least one of the transition states of cyclobutane dissociation (blue and red curves). Because of the high strain-free activation free energy of dissociation of cyclobutane, it is likely to be suitable for gating a diverse range of mechanochemical reactions up to nN-range forces.

**Figure 6:**
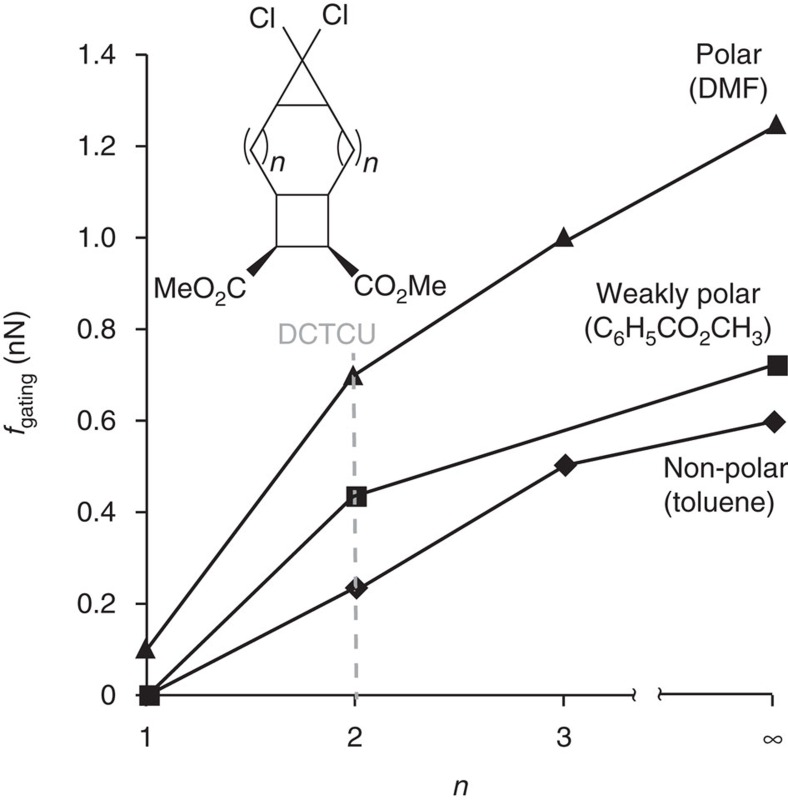
Gating as a function of intervening ring size. The calculated *f*_gating_ (see text for assumptions and computational details) depends on solvent polarity and the size of the separating ring, *n*. Data for *n*=∞ corresponds to a central ring large enough to impose no compressive strain in corresponding TS1 (green structure, Fig. 1a) and was extrapolated using force-dependent activation free energy of ring opening of *cis*-1,1-dichloro-2,3-dimethylcyclopropane.

Second, gating requires that the rate-determining activation barrier of dissociation of the gating moiety be less than the activation barrier of the unloaded substrate. Otherwise, the unloaded substrate will react preferentially, as happens in DCTCU at applied force <0.4 nN (in methylbenzoate). In DCTCU this minimum gating force, *f*_gating_, is determined by the difference of the kinetic barriers for dissociation of *g*DCC and of cyclobutane (that is, TS1 versus the highest of TS1f or TS2f). This difference is sensitive to both the reaction solvent and the size of the ring separating the cyclobutane and *g*DCC moieties, because these variables affect the energy of TS1 but not of TS1f or TS2f. Polar solvents stabilize TS1 (for example, its energy is 39.8 kcal mol^−1^ and 47.9 kcal mol^−1^ in DMF and toluene, respectively), whereas smaller rings destabilize it, by imposing a larger compressive strain on it (for example, shrinking the central ring of DCTCU by 2 atoms to yield 4,4-dichlorotricyclo(5.2.0.0)nonane, *n*=1, Fig. 6 increases TS1 by 12.5 kcal mol^−1^ in toluene). By varying these two parameters, *f*_gating_ can be changed systematically from 0 nN up to ∼1.3 nN, as may be required for a particular application of the gated mechanophore. For example, in DCTCU (*n*=2, Fig. 6), changing the reaction solvent from toluene to DMF increases *f*_gating_ from 0.2 to 0.7 nN. A very large ring separating the *g*DCC and cyclobutane moieties (*n*>3) would shift this range up to 0.6–1.3 nN.

Lastly, force also affects the geometry of the 3 C=C bonds generated by ring opening of DCTCU. The geometry of the central C=C bond is determined by whether the *g*DCC or cyclobutane ring opened first: at force below *f*_gating_, the reaction proceeds through TS1, yielding the *E* isomer, whereas at higher forces *g*DCC isomerizes through TS3f, producing the Z configuration. The geometries of the 2 C=C bonds generated by dissociation of cyclobutane are determined by the relative energies of the conformers of TS2f, all of which are accessible by low-barrier rotation around the two exocyclic bonds of Int1f: at force <0.15 nN, the *EE* conformer is lowest-energy, while the *EZ* conformer dominates at higher forces (Supplementary Fig. 2). Consequently, depending on applied force, DCTCU yields one of the three isomeric trienes as the major product: *EEE* at 0–0.15 nN; *EEZ* at 0.15–0.4 nN (in methylbenzoate) and *EZZ* at higher force. While the generation of the *EZZ* product at 2+ nN is consistent with our experimental results discussed above, as well as the previously reported data for mechanochemical isomerization of bicyclo(4.2.0)octane derivatives^34^, the very high activation barriers of isomerization at forces <0.4 nN have so far prevented us from confirming the calculated mechanism at low forces.

In conclusion, we designed a mechanical gating system, in which one mechanophore functions as a gate to modulate the reactivity of another mechanophore, resulting in a mechanochemical cascade reaction. Both SMFS and sonication results are consistent with the sequential activation. The concept of mechanical gating can be applied to situations where controlled reactivity is desired, including catalysis, small molecule release and stress sensing. In addition, we quantified the mechanochemical kinetics of the cycloreversion of the increasingly popular cyclobutane mechanophore motif^60^,^61^,^62^, and therefore expanded the small database of quantitative measures of mechanochemical reactivity.

## Methods

### SMFS

On a piece of 1 × 1 cm^2^ silicon substrate surface was added 20 μl 0.5 mg ml^−1^ THF solution of the polymer, and the surface was left to dry, allowing the polymer to be absorbed on the surface. The substrate was mounted on the top of a piezo scanner, and the AFM tip was brought into contact with the surface and then retracted from it. The tip velocity was set at 300 nm s^−1^ and the approaching/retracting cycles were recorded. The experiments were conducted in both methyl benzoate and toluene, in different days with different cantilever tips.

### QM calculation

Free energies in Fig. 2 were calculated at the uMPW1K/6-311+G(d) level of the DFT in CPCM model of the reaction solvent using complete conformational ensembles in pseudo-harmonic approximation^35^,^63^. The model chemistry was chosen because it reproduced the experimental free energies of activation of isomerization of *cis*-2,3-dimethyl-1,1-dichlorocyclopropane in DMF and diphenyl ether, and of the electronic activation energies of dissociation of *cis* cyclobutane-1,2-dicarboxylic acid and isomerization of *cis*-2,3-dimethyl-1,1-dichlorocyclopropane in vacuum calculated at the uCCSD/6-31G(d)//uCCSD/jun-cc-pVTZ and uCCSD/6-311+G(d)//uCCSD(T)/jun-cc-pVTZ levels of theory, respectively (Supplementary Tables 1 and 4). Force–extension curves were calculated using the previously reported approach^37^ by combining the force-dependent activation free energies and force–extension correlations of a single repeat unit of polymer **6** (blue curves, Fig. 4) or 2 repeat units of poly(*cis*-*g*DCC) (green curves) in the reactant and the product states, calculated using partial conformational ensembles of 3 lowest-energy conformers for each state. The loading rate used to calculate the force–extension curves was 2–6 nm s^−1^ (depending on the length of the polymer in the corresponding experiment), to reproduce the time that the plateau was traversed in SMFS experiments. The calculated force–extension curves were then scaled to reproduce the shape of the experimental curves between 0.3 and 1.6 nN (for polymer **6**) or between 0.2 and 1 nN (for poly(*cis-g*DCC)).

### Data availability

The data sets generated during and/or analysed during the current study are available from the corresponding authors on reasonable request.

## Additional information

**How to cite this article:** Wang, J. *et al*. Mechanical gating of a mechanochemical reaction cascade. *Nat. Commun.* **7,** 13433 doi: 10.1038/ncomms13433 (2016).

**Publisher's note:** Springer Nature remains neutral with regard to jurisdictional claims in published maps and institutional affiliations.

## Supplementary Material

Supplementary Information Supplementary Figures 1-9, Supplementary Tables 1-4, Supplementary Methods and Supplementary References
