# Supplementary material for: Mechanical gating of a mechanochemical reaction cascade
Source: Nat Commun. 2016 Nov 16;7:13433. doi: 10.1038/ncomms13433 (PMC5116086; doi:10.1038/ncomms13433)
Supplement: Supplementary Information — Supplementary Figures 1-9, Supplementary Tables 1-4, Supplementary Methods and Supplementary References [file ncomms13433-s1.pdf]

Supplementary Figures

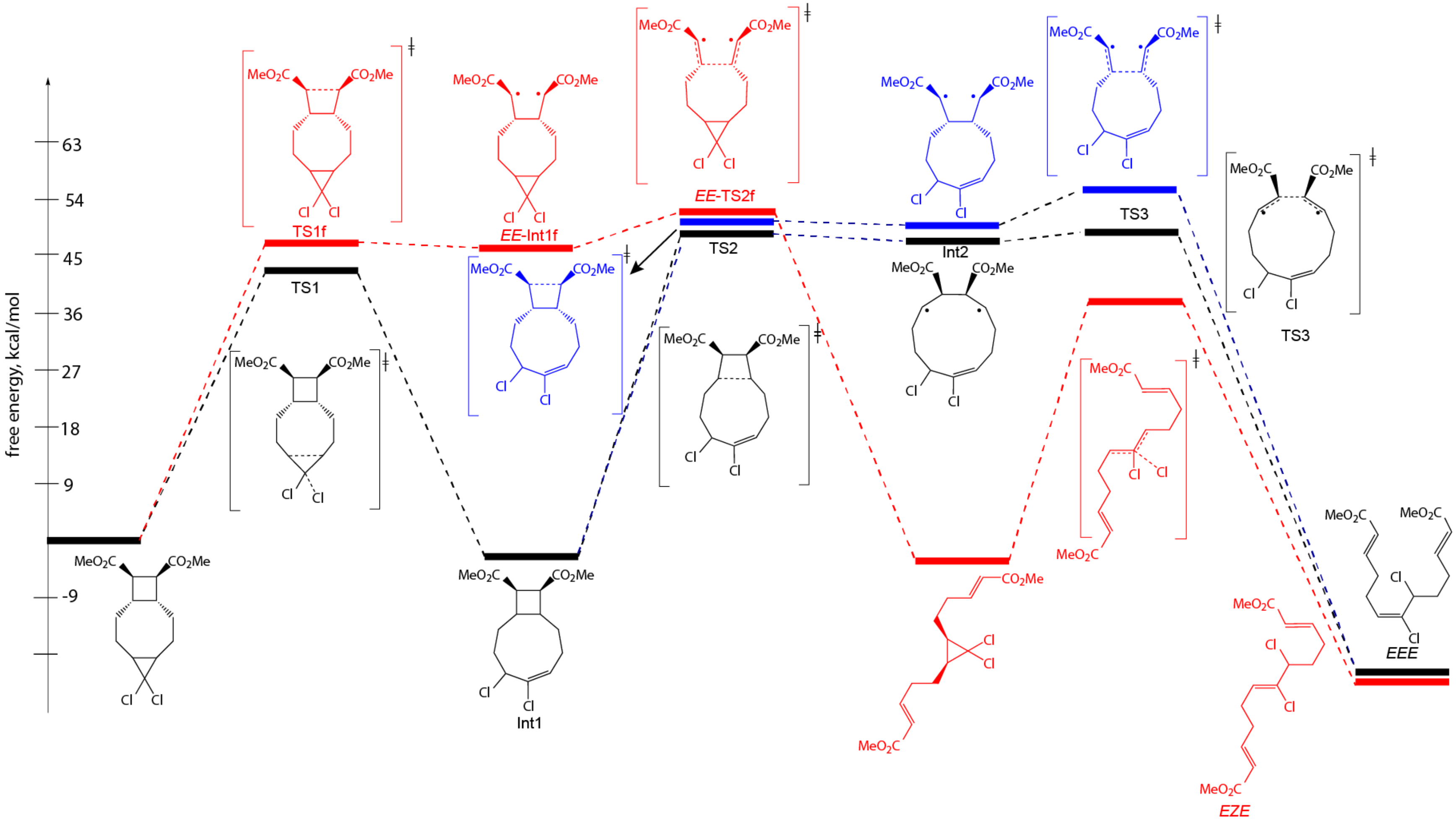

Supplementary Figure 1. Force free reactivity of DCTCU. Free energy diagram for isomerization of DCTCU in the absence of force at the uMPW1K/6-311+G(d) level in toluene-parameterized CPCM model of the reaction solvent. The minimum-energy path (black) has the apparent activation free energy 1.6 and 1.9 kcal/mol lower than the alternatives (blue and red, respectively) under standard conditions. We were unable to locate a transition state corresponding to the dissociation of the inner scissile C-C bond of the cyclobutane moiety in DCTCU.

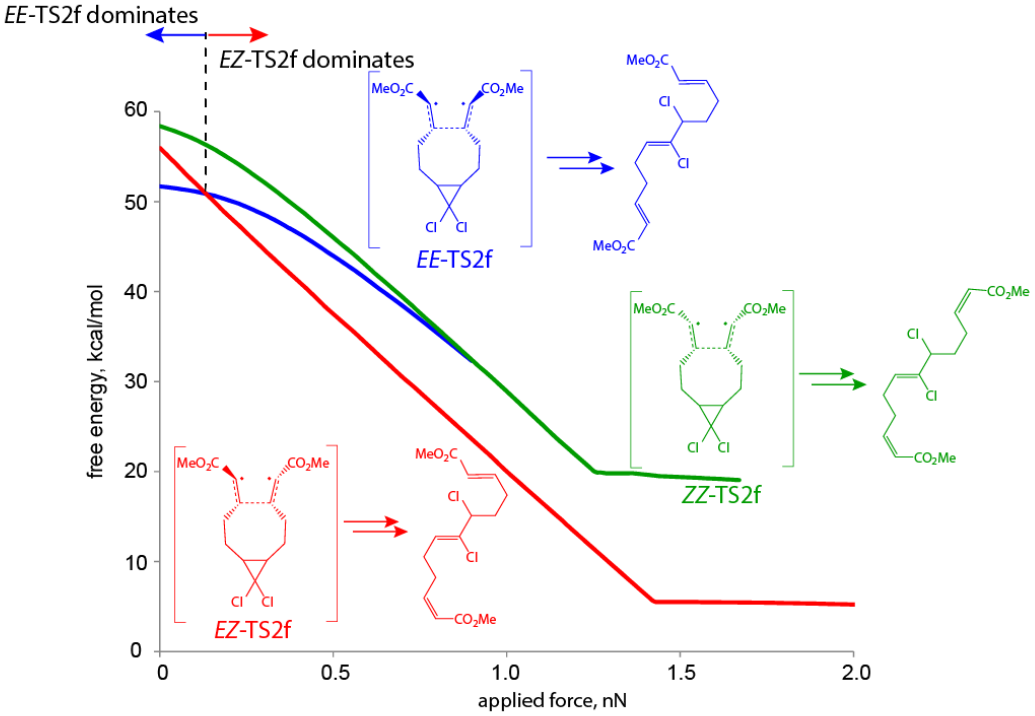

Supplementary Figure 2. Free energies of the *EE*, *EZ* and *ZZ* conformers of TS2f. Energies calculated as a function of applied force. The *EE* conformer dominates at < 0.13 nN and the *EZ* analog dominates at higher force.

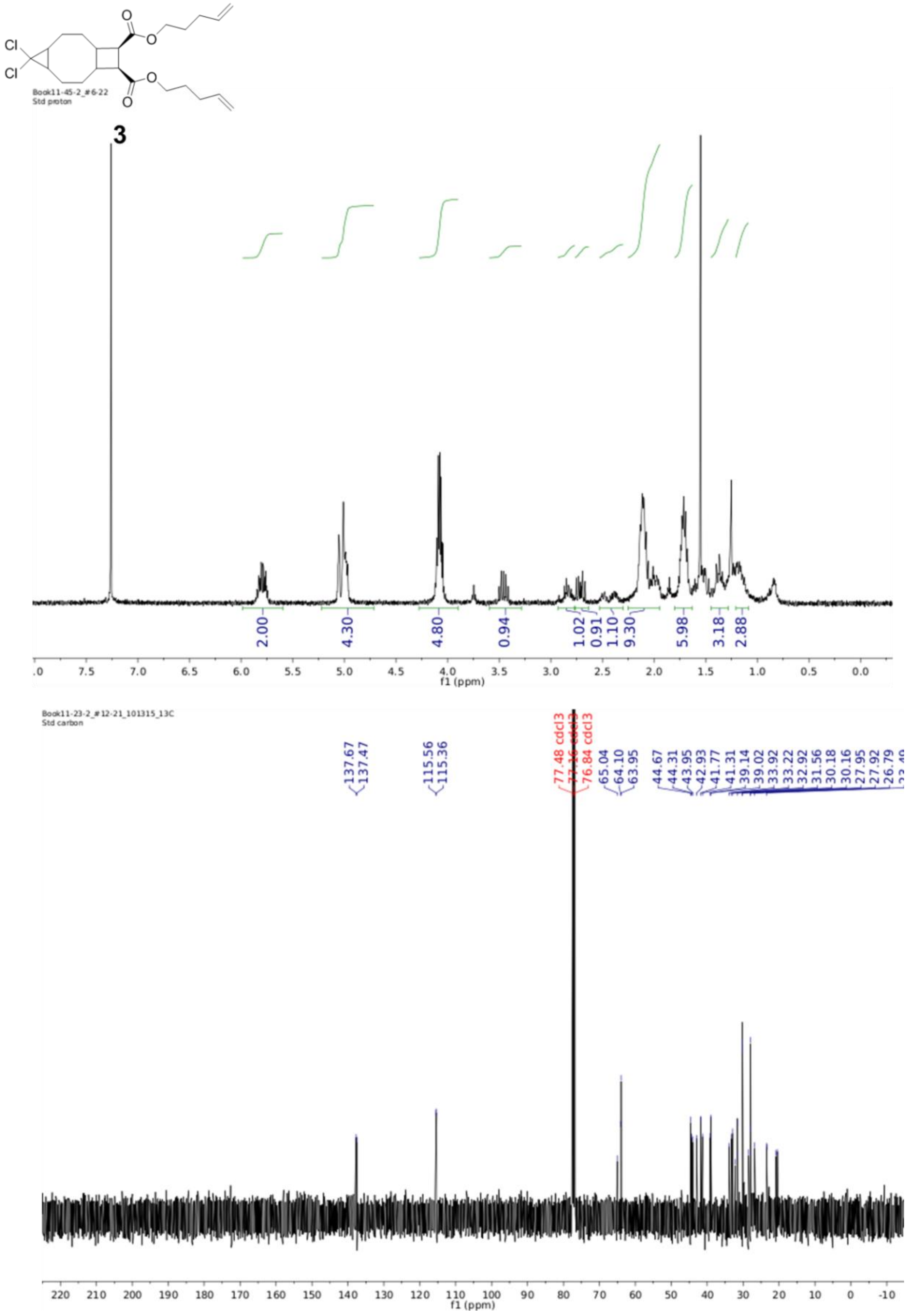

Supplementary Figure 3. NMR spectra of the synthetic intermediates and final products.

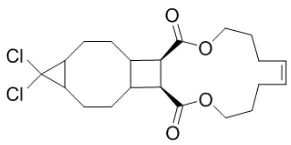

4

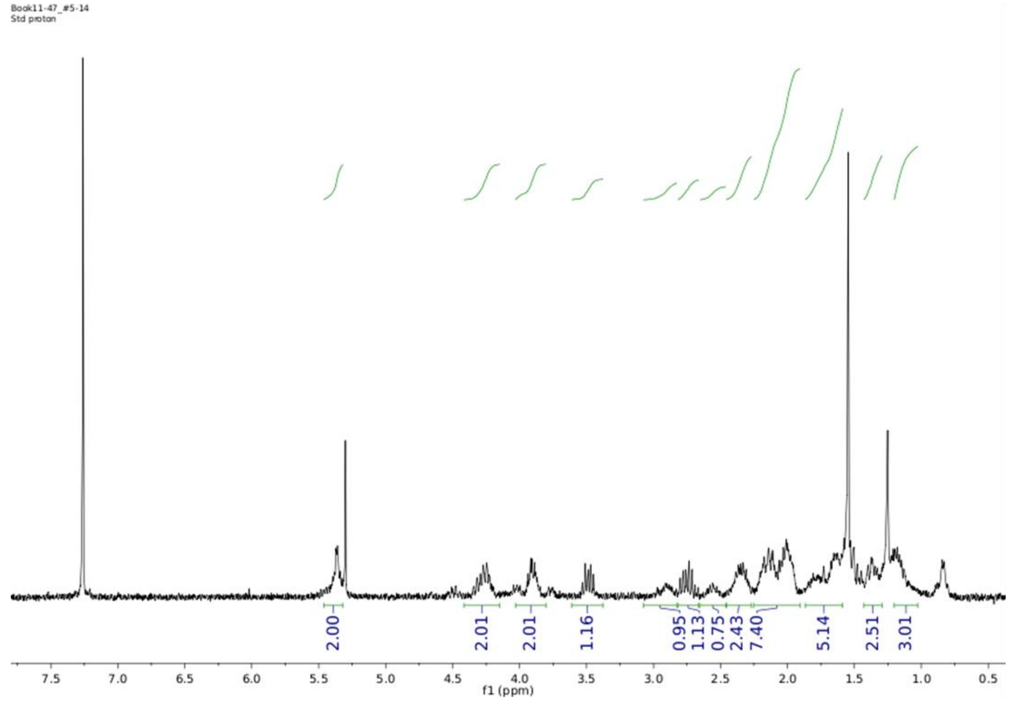

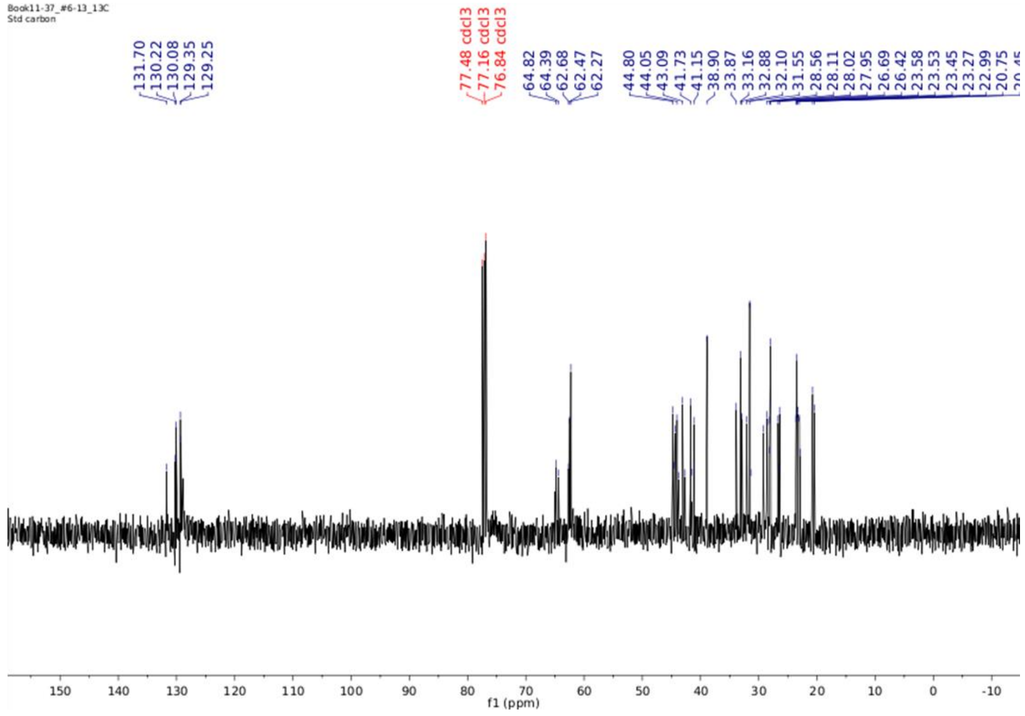

Supplementary Figure 3 (cont'd). NMR spectra of the synthetic intermediates and final products.

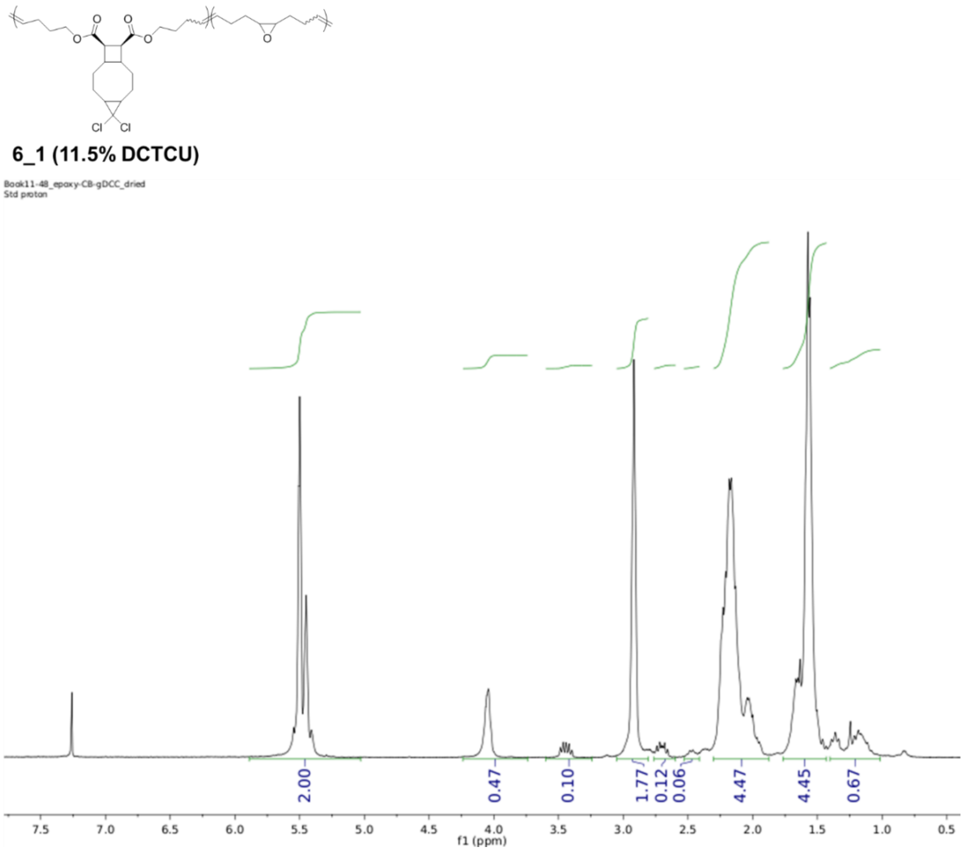

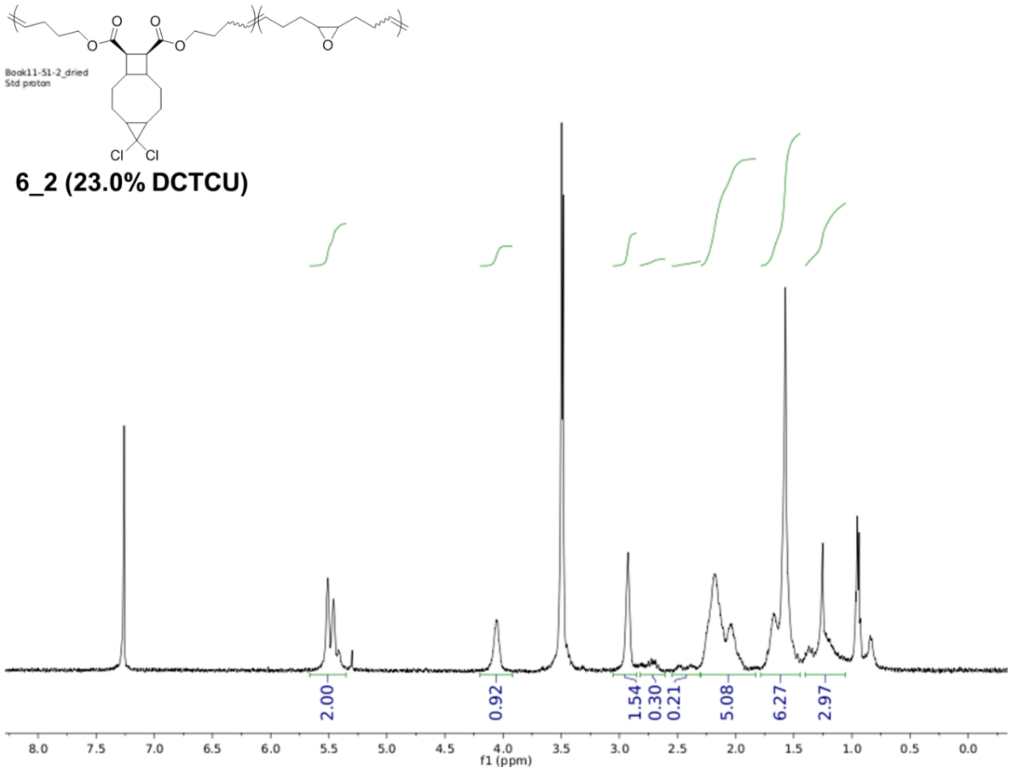

Supplementary Figure 3 (cont'd). NMR spectra of the synthetic intermediates and final products.

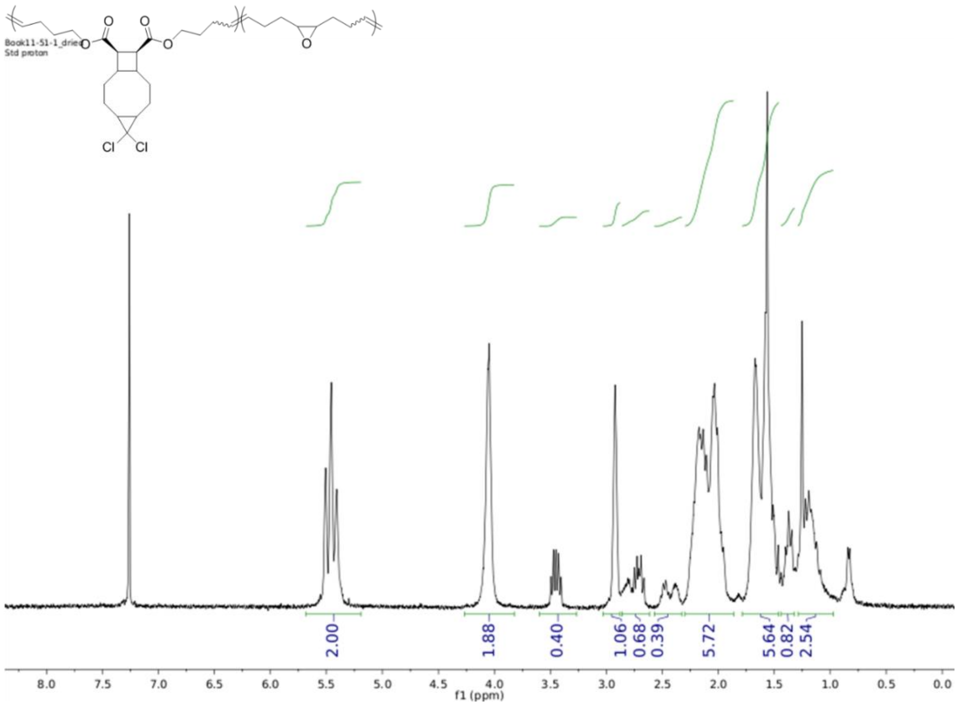

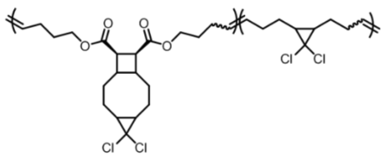

8

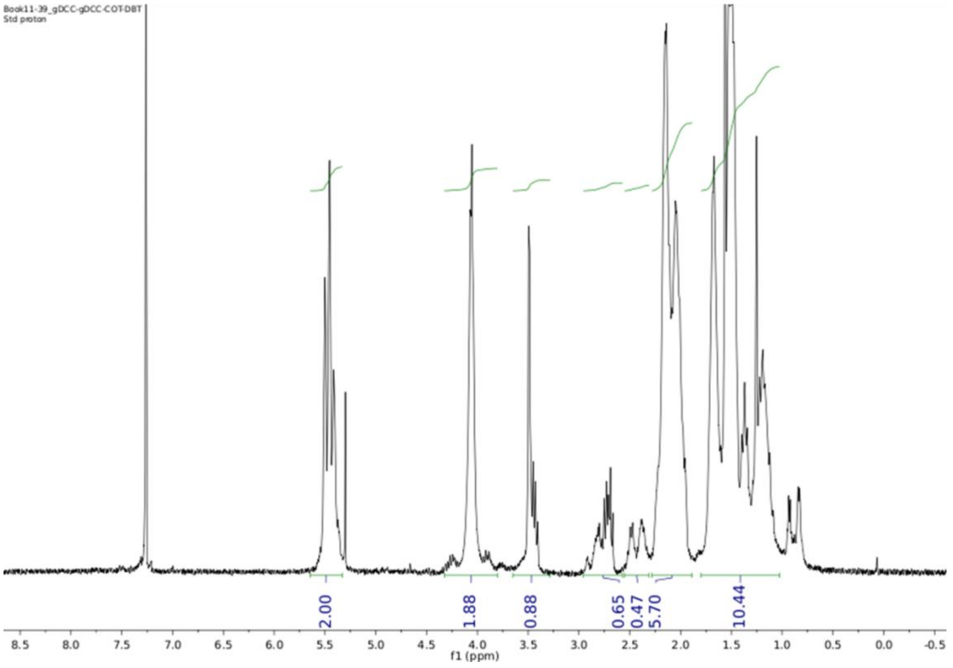

Supplementary Figure 3 (cont'd). NMR spectra of the synthetic intermediates and final products.

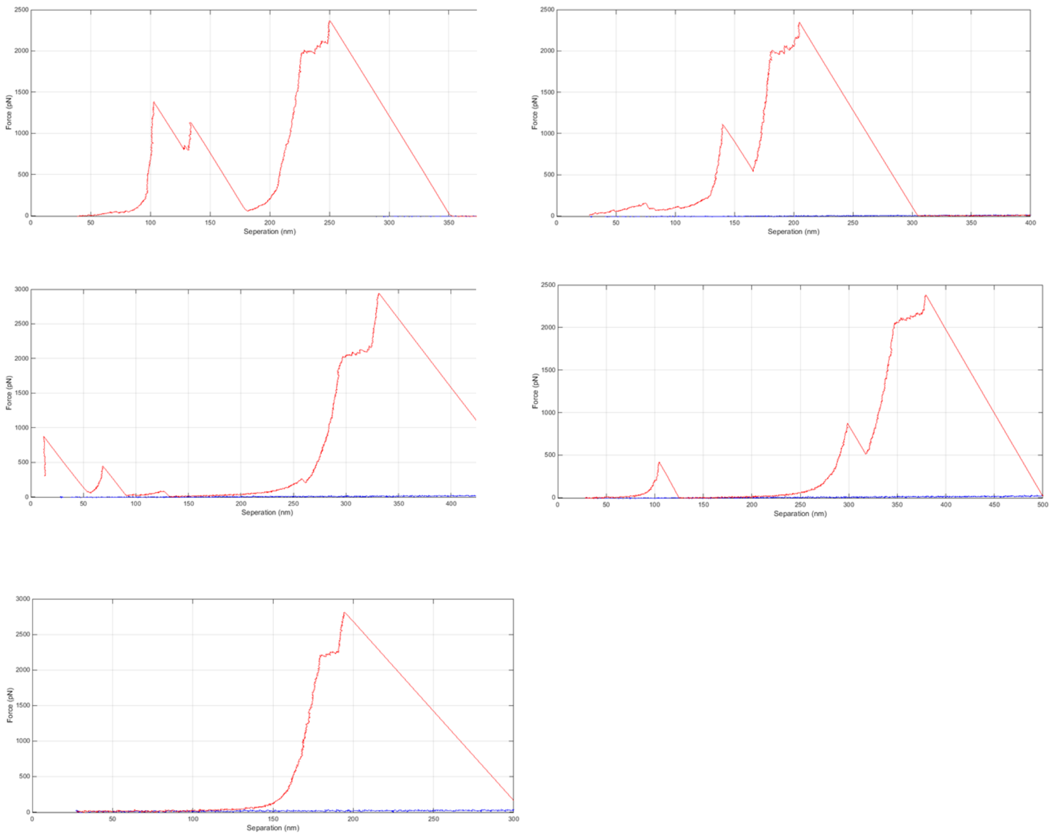

Supplementary Figure 4. Complete force curves of 6_1 (12% DCTCU).

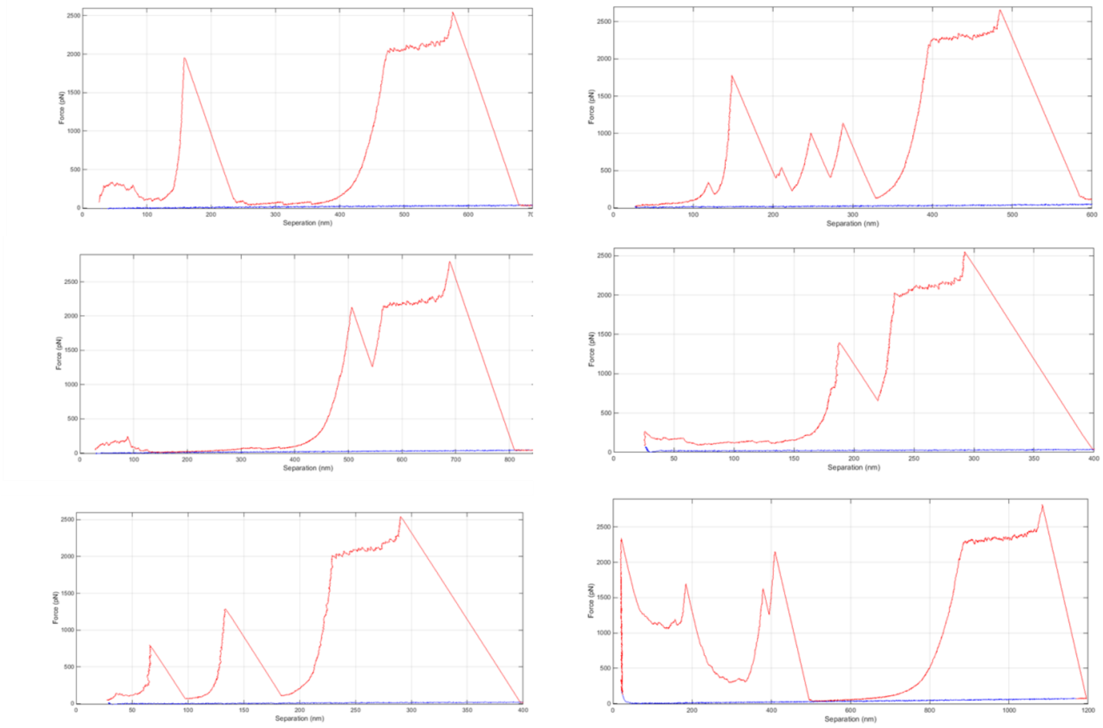

Supplementary Figure 5. Complete force curves of 6_2 (23% DCTCU).

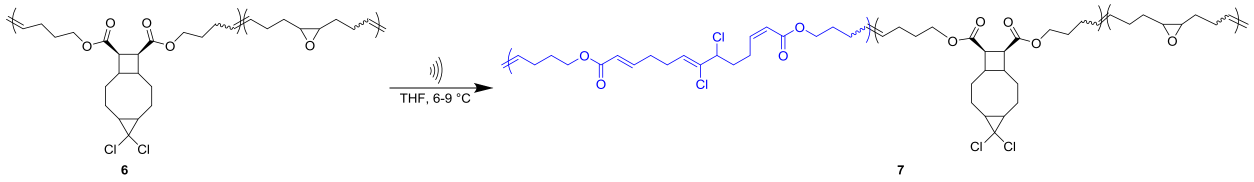

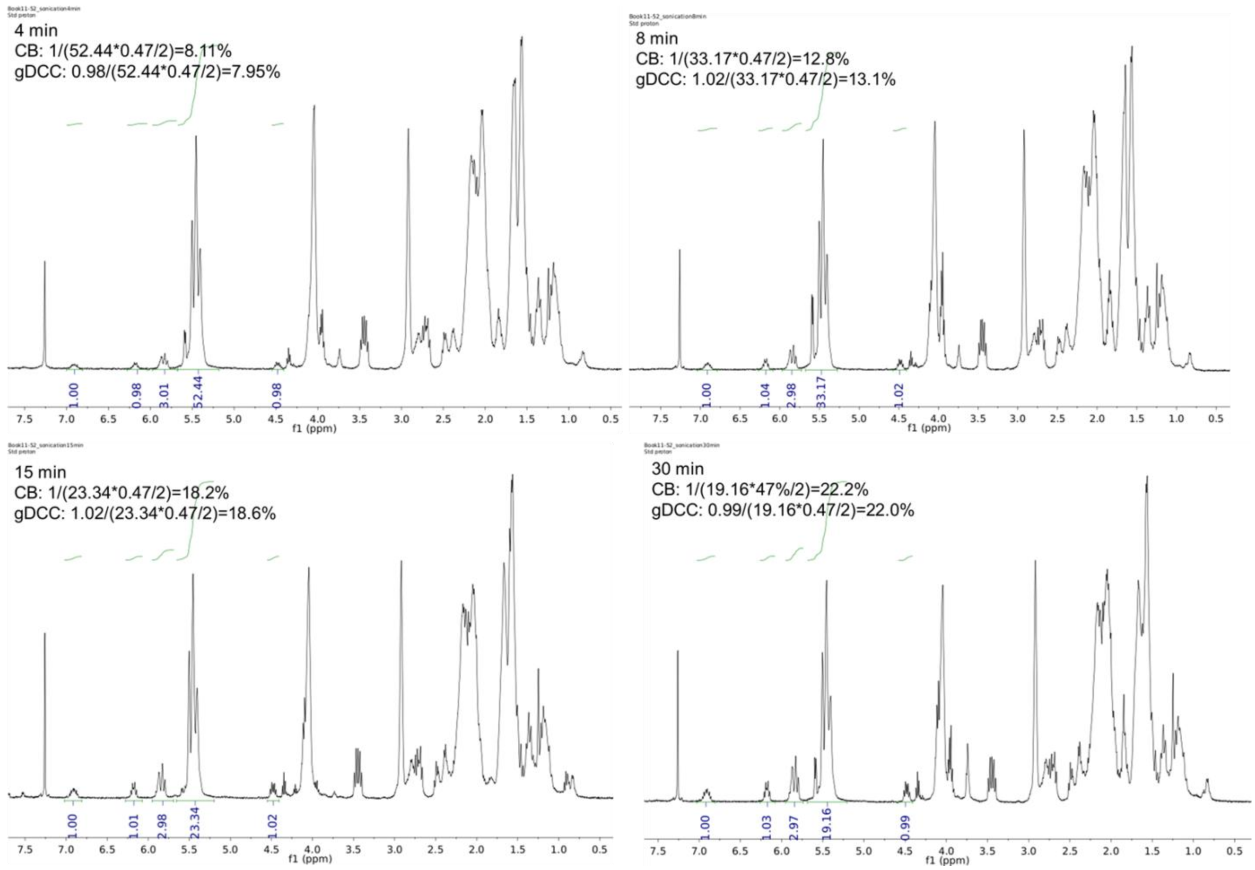

Supplementary Figure 6. Sonication of 6_3 (47% DCTCU). ^{1}H NMR spectra obtained after sonicating polymer **6_3** for 4 min, 8 min, 15 min and 30 min, respectively. The peaks at 6.9 ppm and 6.2 ppm correspond to the *β*-H of the *E*-alkene and *Z*-alkene of the unsaturated esters, respectively; the peak at 5.8 ppm corresponds to the *α*-H of both *E*-alkene and *Z*-alkene of the unsaturated ester as well as the alkene-H of the 2,3-dichloroalkene; the peak at 4.5 ppm corresponds to the allylic-H of the 2,3-dichloroalkene. Integration of the peaks shows that 1) during sonication, the content of *E*-alkene ester is identical to that the *Z*-alkene ester; 2) the content of 2,3-dichloroalkene is identical to that of either *E*- or *Z*- alkene ester. The ring opening fraction for *cis*-*g*DCC is therefore identical to that of cyclobutane.

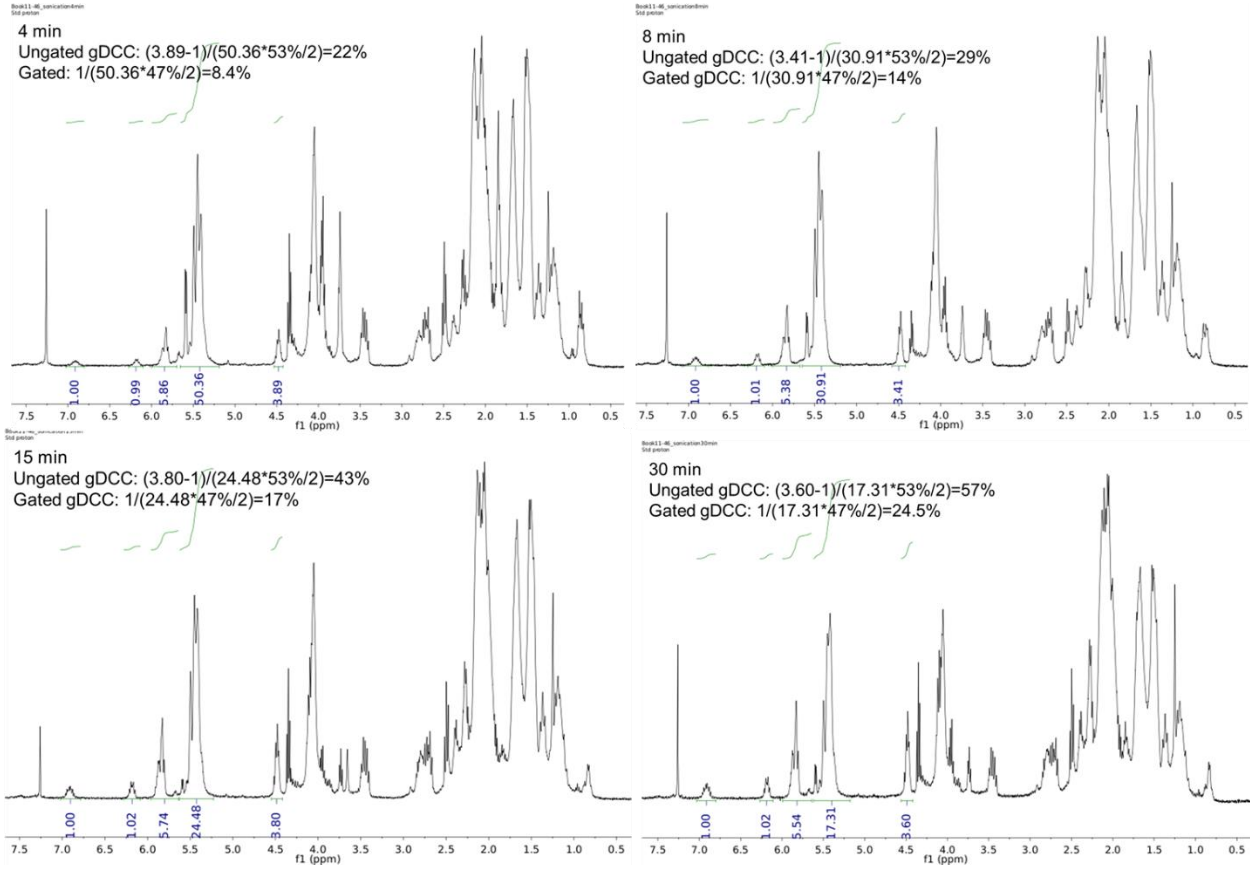

Supplementary Figure 7. Sonication of 8 (53% *cis*-*g*DCC on the backbone, 47% DCTCU). ^{1}H NMR spectra obtained after sonicating polymer **8** for 4 min, 8 min, 15 min and 30 min, respectively. The corresponding ring opening percentages were calculated based on the integrations and shown on the spectra. The ring opening of gated *g*DCC, as shown in Figure 4, is equivalent to the scission of the cyclobutane. The ring opening of the ungated *g*DCC was derived by subtracting the ring opening of the gated *g*DCC from the total ring opening of *g*DCC.

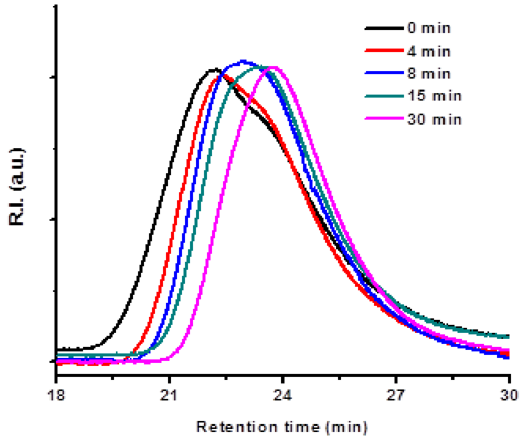

Supplementary Figure 8. Gel-permeation chromatographs of a solution of polymer 6_3. With increasing sonication time, increased retention time is detected using a refractive index detector.

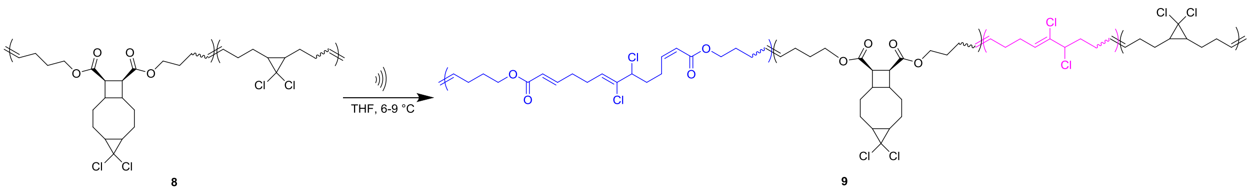

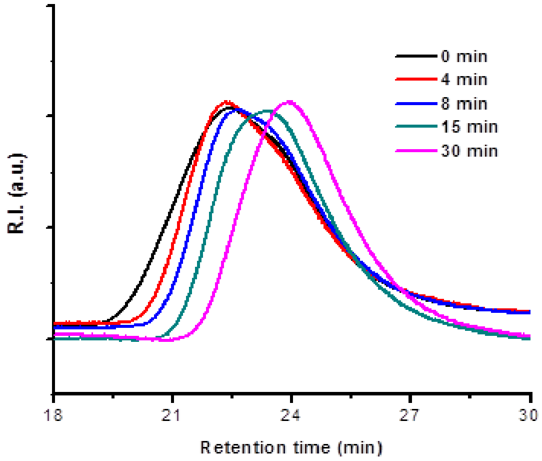

Supplementary Figure 9. **Gel-permeation chromatographs of a solution of polymer 8.** Longer retention times with increasing sonication time indicates molecular mass degradation.

Supplementary Tables

Supplementary Table 1. Computational methods benchmarks. Comparison of the activation energies (in kcal/mol) of *cis*-2,3-dimethyl-1,1-dichlorodimethylcyclopropane isomerization in vacuum, diphenyl ether and DMF from experiment or ab initio calculations with those calculated at the uMPW1K/6-311+G(d) and uB3LYP/6-311+G(d) level of DFT.

	Experimental or uCCSD(T) energy	uMPW1K	uB3LYP
vacuum[2]	52.1[1]	50.2	39.1
Diphenyl ether[3]	37.3	38.8[4]	26.6[4]
DMF[3]	33.5	32.8[4]	24.7[4]
Average error		0.3	10.8

1. uCCSD/6-311+G(d)//uCCSD(T)/jun-cc-pVTZ
2. electronic energy
3. Experimental $\Delta G^{\ddagger}$ at 152 ^{o}C obtained from the Eyring equation.
4. in CPCM parameterized for the corresponding solvent; the thermodynamic correction at the ideal gas/rigid rotor/pseudo-harmonic oscillator as per Kim Y., Mohrig J. R., Truhlar D. G. Free-energy surfaces for liquid-phase reactions and their use to study the border between concerted and nonconcerted α,β-elimination reactions of esters and thioesters. J. Am. Chem. Soc. 2010, 132, 11071 and Cramer, C. J. Essentials of computational chemistry. 2nd ed. edn, p. 355 (Wiley, 2004).

Supplementary Table 2. Key parameters of the measured force-extension curves for polymer 6.

mole fraction of DCTCU	Polymer length,[1] nm	Force at beginning of plateau, nN	Force at end of plateau, nN	Plateau height, pN	Plateau length, nm	# of DCTCU units
0.23	463	2.24	2.40	160	82	70
0.23	662	2.12	2.27	150	113	96
0.23	264	2.00	2.15	150	53	45
0.23	268	2.00	2.20	200	55	47
0.23	1014	2.25	2.41	160	188	160
0.23	556	2.02	2.20	180	94	80
0.12	309	2.04	2.13	90	28	24
0.12	360	2.00	2.15	150	28	24
0.12	173	2.00	2.10	100	13	11
0.12	228	2.17	2.24	70	21	18

1. averaged over 2420-2470 pN

Supplementary Table 3. Calculated force-extension curves of DCTCU-containing polymer **6** and poly(*cis*-*g*DCC). The data tabulated here is plotted in Fig. 4 of the main text.

Force, pN	Polymer 6	Poly(*cis*-*g*DCC)	Force, pN	Polymer 6	Poly(*cis*-*g*DCC)	Force, pN	Polymer 6	Poly(*cis*-*g*DCC)
0	282.0	224.3	1000	364.5	290.6	2000	380.3	334.2
10	299.2	227.2	1010	364.7	290.8	2010	380.4	
20	303.6	230.1	1020	364.9	291.0	2020	380.6	
30	308.0	233.0	1030	365.1	291.1	2030	380.7	
40	312.3	235.9	1040	365.3	291.3	2040	380.8	
50	316.6	238.7	1050	365.5	291.5	2050	380.9	
60	318.5	241.6	1060	365.7	291.7	2060	381.0	
70	320.4	244.5	1070	365.9	291.9	2070	381.2	
80	322.3	247.4	1080	366.1	292.0	2080	381.3	
90	324.2	250.3	1090	366.3	292.2	2090	381.4	
100	326.1	253.1	1100	366.5	292.4	2100	381.6	
110	327.2	254.2	1110	366.7	292.6	2110	381.7	
120	328.2	255.3	1120	366.9	292.8	2120	381.8	
130	329.3	256.4	1130	367.1	292.9	2130	381.9	
140	330.3	257.6	1140	367.3	293.1	2140	382.1	
150	331.4	258.7	1150	367.5	293.3	2150	382.2	
160	332.4	259.8	1160	367.7	293.5	2160	382.4	
170	333.5	260.9	1170	367.9	293.7	2170	382.5	
180	334.5	262.1	1180	368.1	293.9	2180	382.6	
190	335.6	263.2	1190	368.3	294.0	2190	382.8	
200	336.6	264.3	1200	368.5	294.2	2200	382.9	
210	337.4	264.9	1210	368.6	294.4	2210	383.1	
220	338.1	265.5	1220	368.8	294.5	2220	383.2	
230	338.8	266.1	1230	368.9	294.7	2230	383.4	
240	339.5	266.7	1240	369.1	294.9	2240	383.6	
250	340.3	267.3	1250	369.2	295.1	2250	383.8	
260	341.0	267.9	1260	369.4	295.2	2260	384.0	
270	341.7	268.5	1270	369.5	295.4	2270	384.2	
280	342.4	269.0	1280	369.7	295.6	2280	384.5	
290	343.2	269.6	1290	369.8	295.9	2290	384.9	
300	343.9	270.2	1300	370.0	296.1	2300	385.4	
310	344.4	270.6	1310	370.1	296.4	2310	386.4	
320	344.9	271.0	1320	370.3	296.7	2320	392.9	
330	345.3	271.4	1330	370.5	297.2	2330	403.5	
340	345.8	271.8	1340	370.6	298.5	2340	412.6	
350	346.3	272.2	1350	370.8	303.1	2350	420.5	
360	346.8	272.6	1360	370.9	307.5	2360	427.3	
370	347.3	273.0	1370	371.1	311.2	2370	433.2	
380	347.8	273.4	1380	371.3	314.2	2380	438.2	
390	348.2	273.8	1390	371.4	316.7	2390	442.6	
400	348.7	274.2	1400	371.6	318.8	2400	446.4	
410	349.1	274.5	1410	371.7	320.4	2410	449.6	
420	349.5	274.8	1420	371.9	321.7	2420	452.3	
430	349.8	275.2	1430	372.0	322.9	2430	454.8	
440	350.2	275.5	1440	372.2	323.9	2440	456.8	
450	350.6	275.9	1450	372.4	324.7	2450	458.7	
460	351.0	276.2	1460	372.5	325.4	2460	460.3	
470	351.3	276.6	1470	372.7	326.0	2470	461.7	
480	351.7	276.9	1480	372.8	326.5	2480	462.9	
490	352.1	277.2	1490	373.0	327.0	2490	464.0	
500	352.4	277.6	1500	373.1	327.3	2500	464.9	
510	352.7	277.8	1510	373.3	327.7	2510	465.6	
520	353.0	278.1	1520	373.5	328.0	2520	466.3	
530	353.3	278.4	1530	373.6	328.2	2530	466.9	
540	353.6	278.6	1540	373.8	328.5	2540	467.5	
550	353.9	278.9	1550	373.9	328.7	2550	468.0	
560	354.1	279.1	1560	374.1	328.9	2560	468.4	
570	354.4	279.4	1570	374.3	329.0	2570	468.8	

580	354.7	279.6	1580	374.4	329.2	2580	469.2	
590	355.0	279.9	1590	374.6	329.3	2590	469.5	
600	355.3	280.2	1600	374.7	329.5	2600	469.8	
610	355.6	280.4	1610	374.9	329.6	2610	470.0	
620	355.9	280.7	1620	375.1	329.7	2620	470.3	
630	356.2	280.9	1630	375.2	329.8	2630	470.5	
640	356.5	281.2	1640	375.4	329.9	2640	470.7	
650	356.7	281.4	1650	375.5	330.1	2650	470.9	
660	357.0	281.7	1660	375.7	330.2	2660	471.1	
670	357.3	282.0	1670	375.9	330.3	2670	471.2	
680	357.6	282.2	1680	376.0	330.4	2680	471.4	
690	357.9	282.5	1690	376.2	330.5	2690	471.5	
700	358.2	282.7	1700	376.4	330.7	2700	471.7	
710	358.5	283.0	1710	376.5	330.8	2710	471.8	
720	358.7	283.3	1720	376.7	330.9	2720	472.0	
730	358.9	283.6	1730	376.8	331.0	2730	472.1	
740	359.2	283.9	1740	377.0	331.1	2740	472.2	
750	359.4	284.2	1750	377.1	331.2	2750	472.3	
760	359.6	284.5	1760	377.2	331.4	2760	472.5	
770	359.9	284.8	1770	377.4	331.5	2770	472.6	
780	360.1	285.1	1780	377.5	331.6	2780	472.7	
790	360.3	285.4	1790	377.7	331.7	2790	472.8	
800	360.6	285.7	1800	377.8	331.8	2800	472.9	
810	360.8	286.0	1810	378.0	332.0	2810	473.1	
820	361.0	286.3	1820	378.1	332.1	2820	473.2	
830	361.2	286.6	1830	378.2	332.2	2830	473.3	
840	361.3	286.9	1840	378.3	332.3	2840	473.4	
850	361.5	287.2	1850	378.5	332.4	2850	473.5	
860	361.7	287.5	1860	378.6	332.6	2860	473.6	
870	361.9	287.8	1870	378.7	332.7	2870	473.8	
880	362.1	288.1	1880	378.8	332.8	2880	473.9	
890	362.3	288.4	1890	378.9	332.9	2890	474.0	
900	362.5	288.7	1900	379.1	333.0	2900	474.1	
910	362.7	288.9	1910	379.2	333.1	2910	474.2	
920	362.9	289.1	1920	379.3	333.3	2920	474.3	
930	363.1	289.3	1930	379.4	333.4	2930	474.5	
940	363.3	289.4	1940	379.6	333.5	2940	474.6	
950	363.5	289.6	1950	379.7	333.6	2950	474.7	
960	363.7	289.8	1960	379.8	333.7	2960	474.8	
970	363.9	290.0	1970	379.9	333.9	2970	474.9	
980	364.1	290.2	1980	380.1	334.0	2980	475.0	
990	364.3	290.4	1990	380.2	334.1	2990	475.1	

Supplementary Table 4. Comparison of electronic energies (in kcal/mol) of conformers of the 1st (TS1) and 2nd (TS2) transition states of thermal dissociation of cis-cyclobutane-1,2-dicarboxylic acid at uCCSD/6-31G(d)//uCCSD/jun-cc-pVTZ, uMPW1K/6-311+G(d) and uB3LYP/6-311+G(d) levels of theory. The blue arrows in the structures show the atomic motion comprising the reactive mode.

	uMPW1K/6-311+G(d) geometry	uCCSD/6-31G(d)// uCCSD/jun-cc-pVTZ	uMPW1K/6-311+G(d)	B3LYP/6-311+G(d)
TS1	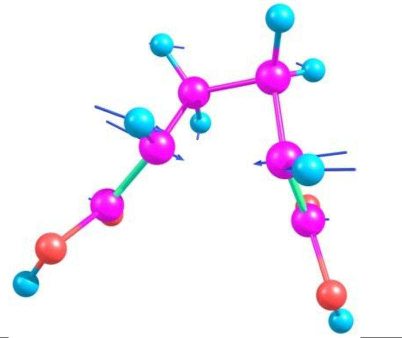	50.8	47.1	43.4
TS2	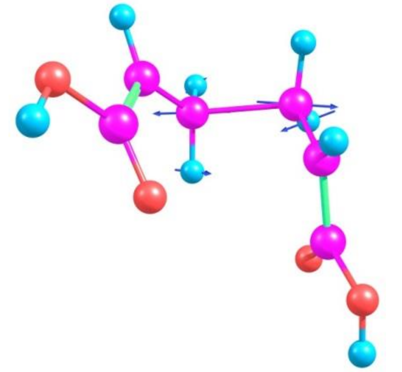	53.4	52.4	42.2
TS2	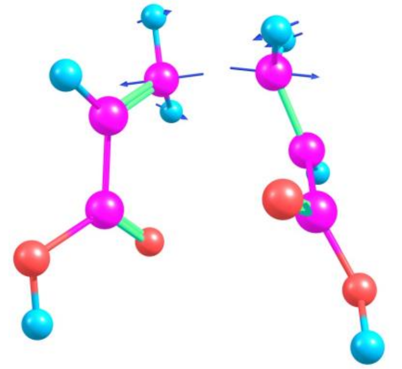	51.8	51.6	42.2
	**Average error**		**1.6**	**9.4**

Supplementary Methods

Unless otherwise mentioned, all starting materials and reagents were purchased from Sigma-Aldrich and used as received. Dry solvents were purchased from VWR. ^{1}H NMR and ^{13}C NMR analysis were conducted on a 400 MHz Varian Spectrophotometer and the residual solvent peaks ($CDCl_3$ 7.26 [^{1}H], 77.16 ppm [^{13}C]) were used as internal chemical shift references. All chemical shifts are given in ppm (δ) and coupling constants (*J*) in Hz as singlet (s), doublet (d), triplet, quartet (q), multiplet (m), or broad (br). Column chromatography was performed using Silicycle F60 (230-400 mesh) silica gel.

Gel permeation chromatography (GPC) was performed on an in-line two columns (Agilent Technology PL gel, 10^4 and 10^3 Å) using THF (HPLC grade, 99.7+%, stabilized with 250 ppm BHT) as eluent at a flow rate of 1 mL min^{-1} under ambient condition. Molecular weights were calculated using a Wyatt Dawn EOS multi-angle light scattering (MALS) detector and Wyatt Optilab DSP Interferometric Refractometer (RI). The refractive index increment (dn/dc) values were determined by online calculation using injections of known concentration and mass.

Single-molecule force spectroscopy (SMFS) measurements were conducted using the same procedure as described previously.[1-3] All of the SMFS studies were conducted at ambient temperature (~23 °C) using a homemade AFM, which was constructed using a Digital Instruments scanning head mounted on top of a piezoelectric positioner, similar to the one described in detail previously.[4] The AFM pulling experiments were conducted in solutions of toluene or methyl benzoate, and no significant difference was observed between the results in the two solvents. The force curves used for analysis were obtained with rectangular-shaped cantilevers (205 μm × 15 μm, nominal tip radius ~2 nm, nominal spring constant k ~ 0.02 N/m, frequency ~15 kHz). The spring constant of each cantilever was calibrated in air, using the MFP-3D system (Asylum Research Group Inc., Santa Barbara, CA), applying the thermal noise method, based on the energy equipartition theorem as described previously.[5] Measurements were carried out in a closed fluid cell with scanning set for a series of approaching/retracting cycles.

The sonication experiment was performed on a Vibracell Model VCX 500 operating at 20 kHz with a 12.8 mm titanium tip probe from Sonics and Materials (http:www.sonics.biz/). Each sonication was performed on 1 mg/mL polymer solution of THF, which was purified with a Pure Solv™ solvent purification system. Prior to sonication, the solution was transferred to a 3-neck Suslick cell in an ice bath and deoxygenated by bubbling through nitrogen for 30 min. Irradiations were performed at 11.9 W/cm^2 with a pulse sequence of 1s on/1s off while maintaining a temperature of 6-9 °C under a nitrogen atmosphere.

Synthesis

Compound 3: Di(pent-4-en-1-yl) (10R,11S)-5,5-dichlorotricyclo[7.2.0.04,6]undecane-10,11-dicarboxylate

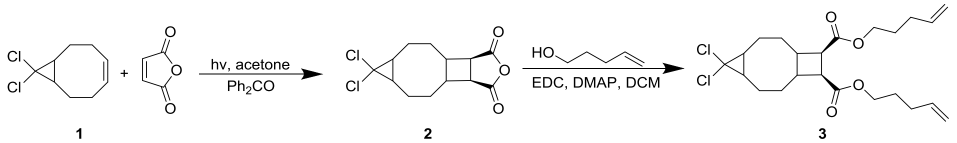

1 (*gem*-dichlorocyclopropane cyclooctene) was made by following previously reported procedure.[6] The [2+2] cycloaddition reaction was performed using similar procedure as the synthesis of Bicyclo[4.2.0]octane.[7] Benzophenone (2.7 g, 15 mmol), maleic anhydride (10.0 g, 102 mmol), and **1** (19.1 g, 100 mmol) were dissolved in 300 mL acetone in a photochemical reactor fitted with a water-cooled quartz emersion well. The solution was sparged with argon for 30 min and then irradiated with a 450 W medium pressure mercury arc lamp through a Pyrex filter for 12 h under argon. Acetone was removed under rotary evaporation and maleic anhydride was removed via vacuum distillation. The residue solid was washed with hexane, dried and used without further purification.

That solid, taken to be intermediate **2** (0.5 g), 4-penten-1-ol (0.387 g, 4.50 mmol), 4-Dimethylaminopyridine (DMAP) (0.211 g, 1.73 mmol) were added to a 100 mL round-bottom flask, and 50 mL dichloromethane (DCM) was added to dissolve the starting materials. The solution was cooled in an ice/water bath before N-(3-Dimethylaminopropyl)-N'-ethylcarbodiimide hydrochloride (EDC) (1.33 g, 6.92 mmol) was added. The resulting solution was left to warm to room temperature and stir overnight. Upon completion of the reaction, the solution was washed 2 times with deionized water and 2 times with brine and dried with Na_2SO_4 (s). The crude product was purified by silica gel column chromatography using 20:1 hexane:ethyl acetate, yielding **3** as a white solid. ^{1}H-NMR (400 MHz, $CDCl_3$) δ 5.90-5.70 (m, 2H), 5.18 – 4.86 (m, 4H), 4.24 – 3.89 (m, 4H), 3.59 – 3.31 (m, 1H), 2.92 – 2.79 (m, 1H), 2.79 – 2.60 (m, 1H), 2.55 – 2.28 (m, 1H), 2.25 – 1.91 (m, 9H), 1.80 – 1.64 (m, 5H), 1.43 – 1.30 (m, 2H), 1.25 – 0.99 (m, 2H); ^{13}C NMR (101 MHz, $CDCl_3$) δ 137.67, 137.47, 115.56, 115.36, 65.04, 64.10, 63.95, 44.67, 44.31, 43.95, 42.93, 41.77, 41.31, 39.14, 39.02, 33.92, 33.22, 32.92, 32.20, 31.61, 31.56, 30.18, 30.16, 28.46, 27.98, 27.95, 27.92, 26.79, 23.49, 23.33, 20.83, 20.47. HRMS (ESI-TOF): calcd for $C_{23}H_{33}Cl_2O_4$ ([MH]+) 443.1750, found 443.1740.

Compound 4: 15,15-dichloro-3,4,5,8,9,10,12b,13,14,14a,15,15a,16,17,17a,17b-hexadecahydro-1H-cyclopropa[5',6']cycloocta[1',2':3,4]cyclobuta[1,2-c][1,6]dioxacyclotetradecine-1,12(12aH)-dione

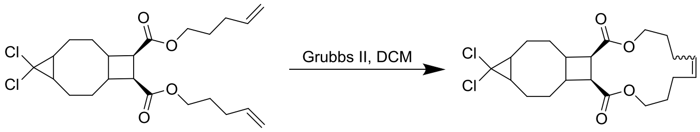

3 4

A flame-dried 250 mL 2-neck round bottom flask was fit with a magnetic stir bar and a condenser. In the flask, **3** (0.47 g, 1.06 mmol) was dissolved in 100 mL DCM. The solution was degassed by bubbling through N_2 for 30 min at room temperature. Grubbs second generation catalyst (0.045 g, 0.053 mmol) was added to the solution under N_2. The reaction was refluxed for 18 h. Upon completion of the reaction, 1 mL ethyl vinyl ether was added to quench the reaction and the mixture was stirred for 30 min. The reaction was cooled to room temperature and concentrated under reduced pressure. The product was purified through column chromatography on silica gel using DCM as the eluent to yield the pure product of **4** (0.3 g, 68%). ^{1}H NMR (400 MHz, $CDCl_3$) δ 5.52 – 5.30 (m, 2H), 4.40 – 4.13 (m, 2H), 4.02 - 3.78 (m, 2H), 3.58 – 3.30 (m, 1H), 3.04 – 2.83 (m, 1H), 2.82 – 2.64 (m, 1H) 2.64 - 2.45 (m, 1H), 2.44 - 2.25 (m, 2H), 2.24 – 1.89 (m, 6H), 1.85 – 1.55 (m, 5H), 1.38 – 1.25 (m, 2H), 1.25-1.01 (m, 3H); ^{13}C-NMR (101 MHz, $CDCl_3$) δ 131.70, 130.22, 130.08, 129.35, 129.25, 64.82, 64.39, 62.68, 62.47, 62.27, 44.80, 44.50, 44.39, 44.05, 43.77, 43.09, 42.66, 41.73, 41.57, 41.15, 41.06, 38.90, 33.87, 33.16, 32.88, 32.10, 31.55, 31.36, 29.24, 28.56, 28.50, 28.19, 28.11, 28.02, 27.95, 26.69, 26.55, 26.42, 23.58, 23.53, 23.45, 23.27, 22.99, 22.89, 20.75, 20.45. HRMS (ESI-TOF): calcd for $C_{21}H_{28}Cl_2O_4$ ([MH]+) 415.1437, found 415.1435.

6: copolymer of DCTCU and epoxide

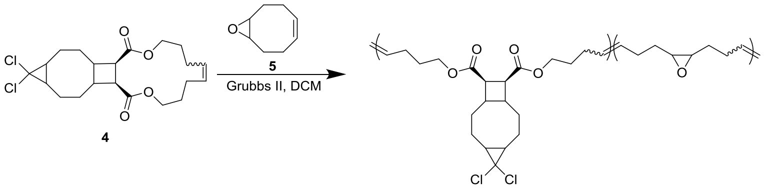

4 6

6_1 (12% DCTCU)

0.04 g **4** (0.097 mmol) and 0.08 g **5**[8] (0.645mmol) were dissolved in 0.1 mL DCM. 1.64 mg (0.00194 mmol) Grubbs second generation catalyst was dissolved in 1 mL DCM. 0.1 mL of the Grubbs catalyst solution was transferred to the monomer solution via a syringe. The viscosity of the solution increased after 30 min and stirring ceased quickly. 0.1 mL of DCM was added to the solution to allow the stirring to continue and the reaction was allowed to proceed overnight. The reaction was quenched with 1 mL of ethyl vinyl ether and stirred for 1 h. The reaction was then precipitated in methanol, redissolved in DCM

and reprecipitated in methanol and dried on a vacuum line. ^{1}H-NMR (400 MHz, $CDCl_3$) δ 5.88 – 5.00 (m, 2H), 4.25 – 3.81 (m, 0.47H), 3.57 – 3.31 (m, 0.1H), 3.11 – 2.77 (m, 1.77H), 2.77 -2.57 (m, 0.1H), 2.55-2.31 (m, 0.1H), 2.47 – 1.82 (m, 4.5H), 1.77 – 1.41 (m, 4.5H), 1.30 -1.02 (m, 0.67H). Copolymer composition: 11.5% DCTCU, 88.5% epoxides. M_n = 102,000, PDI = 1.66, dn/dc = 0.10 mL/g.

6_2 (23% DCTCU)

Similar procedure was used as **6_1**, and the content of DCTCU was controlled by changing the feed ratio. ^{1}H NMR (400 MHz, $CDCl_3$) δ 5.57 – 5.38 (m, 2H), 4.18 – 3.92 (m, 0.9H), 3.08 – 2.82 (m, 1.5H), 2.78 – 2.56 (m, 0.3H), 2.54 – 2.31 (m, 0.2H), 2.33 – 1.86 (m, 5H), 1.80 – 1.44 (m, 6H), 1.40 – 1.03 (m, 3H). Copolymer composition: 23.0% DCTCU, 77.0% epoxides. M_n = 113,100, PDI = 1.43, dn/dc = 0.09 mL/g.

6_3 (47% DCTCU)

Similar procedure was used as **6_1**, and the content of DCTCU was controlled by changing the feed ratio. ^{1}H-NMR (400 MHz, $CDCl_3$) δ 5.69 – 5.24 (m, 2H), 4.24 – 3.87 (m, 1.9H), 3.60 – 3.27 (m, 0.4H), 3.03 – 2.85 (m, 1H), 2.81 – 2.58 (m, 0.7H), 2.55 – 2.31 (m, 0.4H), 2.28 – 1.88 (m, 5.7H), 1.79 – 1.46 (m, 5.6H), 1.44 – 1.31 (m, 0.8H), 1.29 – 0.97 (m, 2.5H). Copolymer composition: 47.0% DCTCU, 53.0% epoxides. M_n = 97,300, PDI = 1.69, dn/dc = 0.07 mL/g.

8: copolymer of DCTCU and *cis-g*DCC

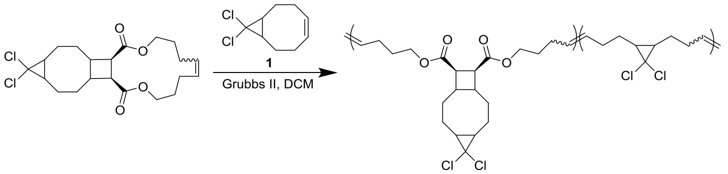

4 8

0.08 g **4** (0.18 mmol) and 0.03 g **1** (0.18 mmol) were dissolved in 0.1 mL DCM. 1.7 mg (0.002 mmol) Grubbs second generation catalyst was dissolved in 1 mL DCM. 0.1 mL of the Grubbs catalyst solution was transferred to the monomer solution via a syringe. The viscosity of the solution increased after 30 min and stirring ceased quickly. 0.1 mL of DCM was added to the solution to allow the stirring to continue and the reaction was allowed to proceed overnight. The reaction was quenched with 1 mL of ethyl vinyl ether and stirred for 1 h. The reaction was then precipitated in methanol, redissolved in DCM and reprecipitated in methanol and dried on a vacuum line. ^{1}H-NMR (400 MHz, $CDCl_3$) δ 5.60 – 5.35 (m, 2H), 4.34 – 3.75 (m, 1.9H), 3.60 – 3.27 (m, 0.9H), 2.94 – 2.60 (m, 0.7H), 2.54 – 2.31 (m, 0.5H), 2.27 – 1.87 (m, 5.7H), 1.78 – 1.02 (m, 10.4H). Copolymer composition: 47.0% DCTCU, 53.0% *cis-g*DCC (backbone). M_n = 97,200, PDI = 1.60, dn/dc = 0.07 mL/g.

Supplementary References

1. Wu, D., Lenhardt, J. M., Black, A. L., Akhremitchev, B. B. & Craig, S. L. Molecular stress relief through a force-induced irreversible extension in polymer contour length. *J. Am. Chem. Soc.* **132**, 15936-15938, (2010).
2. Klukovich, H. M., Kouznetsova, T. B., Kean, Z. S., Lenhardt, J. M. & Craig, S. L. A backbone lever-arm effect enhances polymer mechanochemistry. *Nat. Chem.* **5**, 110-114, (2013).
3. Wang, J. *et al.* Inducing and quantifying forbidden reactivity with single-molecule polymer mechanochemistry. *Nat. Chem.* **7**, 323-327, (2015).
4. Oberhauser, A. F., Marszalek, P. E., Erickson, H. P. & Fernandez, J. M. The molecular elasticity of the extracellular matrix protein tenascin. *Nature* **393**, 181-185, (1998).
5. Florin, E. L. *et al.* Sensing specific molecular-interactions with the atomic-force microscope. *Biosens. Bioelectron.* **10**, 895-901, (1995).
6. Okazaki, R., Ooka, M., Tokitoh, N. & Inamoto, N. Synthesis and reactions of 1,6-dithiocyanato- and 1,6-diiodo-1,3,5-cycloheptatrienes. *J. Org. Chem.* **50**, 180-185, (1985).

7. Kean, Z. S., Niu, Z., Hewage, G. B., Rheingold, A. L. & Craig, S. L. Stress-responsive polymers containing cyclobutane core mechanophores: Reactivity and mechanistic insights. *J. Am. Chem. Soc.* **135**, 13598-13604, (2013).
8. Anderson, R. J. & Henrick, C. A. Stereochemical control in Wittig olefin synthesis - preparation of pink bollworm sex-pheromone mixture, gossyplure. *J. Am. Chem. Soc.* **97**, 4327-4334, (1975).
